# LANGUAGE-BASED DISCRIMINATION RELATES TO MEMORY COMPLAINTS AMONG LATINX/HISPANIC OLDER ADULTS

**DOI:** 10.1093/geroni/igac059.1384

**Published:** 2022-12-20

**Authors:** Elizabeth Soto, Silvia Chapman, Indira Turney, Laura Zahodne, Stephanie Cosentino, Adam Brickman, Jet Vonk, Jennifer Manly

**Affiliations:** Columbia Medical Center, New York City, New York, United States; Columbia University Irving Medical Center, New York City, New York, United States; Columbia University Medical Center, New York, New York, United States; University of Michigan, Ann Arbor, Michigan, United States; Columbia University Medical Center, New York, New York, United States; Columbia Medical Center, New York, New York, United States; Columbia University, New York, New York, United States; Columbia University, New York, New York, United States

## Abstract

Memory Complaints (MCs) are a risk factor for dementia, but research in this area has largely been limited to non-Latinx White adults. Previous studies have shown that Latinx/Hispanic individuals are at higher risk for Alzheimer’s Disease and Related Disorders (ADRD). The mechanisms underlying this disparity in ADRD are multidimensional and can include negative social stressors such as discrimination. Language discrimination can be a source of stress in this population and might be an important contributor to MCs. We examined if MCs varies as a function of language-based discrimination in a total of 495 Latinx/Hispanic older adults without dementia from the community-based Washington Heights Inwood Columbia Aging Project. Language-based discrimination was measured with a dichotomous item that inquired if individuals had been discriminated against because they do not speak English well (yes or no). A linear regression was conducted to examine the cross-sectional association between language-based discrimination and MCs adjusted for age, education, sex/gender, socioeconomic status (income), and depressive symptoms. The experience of language discrimination was independently associated with MCs (B= 0.83, 95% CI=0.13, 1.54, SE= 0.36, p= .021). Results demonstrate a relationship between negative stressors and the expression of MCs. Future studies should comprehensively examine other discriminatory stressors which may also impact the expression of MCs and the risk for progression to ADRD. Identifying language-based and other discriminatory stressors that negatively affect Latinx/Hispanic communities will allow us to develop models that aim to assess and prevent discrimination, as a protective mechanism for the development of MCs and ADRDs.

